# Comparing oncological outcomes of laparoscopic vs open radical nephroureterectomy for the treatment of upper tract urothelial carcinoma: A propensity score-matched analysis

**DOI:** 10.1080/2090598X.2020.1817720

**Published:** 2020-09-04

**Authors:** Marco Moschini, Stefania Zamboni, Luca Afferi, Benjamin Pradere, Mohammad Abufaraj, Francesco Soria, David D’Andrea, Morgan Roupret, Alexandre De la Taille, Claudio Simeone, Agostino Mattei, Romain Mathieu, Karim Bensalah, Manfred Peter Wirth, Francesco Montorsi, Alberto Briganti, Andrea Gallina, Giuseppe Simone, Michele Gallucci, Carlo Di Bona, Giancarlo Marra, Andrea Mari, Ettore Di Trapani, Mario Alvarez Maestro, Wojciech Krajewski, Shahrokh F. Shariat, Evanguelos Xylinas, Philipp Baumeister

**Affiliations:** a Department of Urology, Vienna General Hospital, Medical University of Vienna, Vienna, Austria; b Department of Urology, Luzerner Kantonsspital, Luzern, Switzerland; c Urology Unit, ASST Spedali Civili, Brescia, Italy; d Department of Medical and Surgical Specialties, Radiological Science and Public Health, University of Brescia, Brescia, Italy; e Department of Urology, CHRU Tours, Francois Rabelais University, Tours, France; f Division of Urology, Department of Special Surgery, Jordan University Hospital, the University of Jordan, Amman, Jordan; g Division of Urology, Department of Surgical Sciences, University of Studies of Torino, Turin, Italy; h Urology, Assistance Publique-Hôpitaux De Paris (AP-HP), Sorbonne University, Hopital Pitié Salpétrière, Paris, France; i Department of Urology, Assistance Publique-Hôpitaux De Paris (AP-HP) CHU Mondor, Faculté De Médecine, Henri Mondor Hospital, Créteil, France; j Urology, Rennes University Hospital (France), Rennes, France; k Department of Urology, University Hospital Carl Gustav Carus, Dresden, Germany; l Department of Urology, Urological Research Institute, Vita-Salute University, San Raffaele Scientific Institute, Milan, Italy; m Department of Urology, ‘Regina Elena’ National Cancer Institute, Rome, Italy; n Department of Urology, University of Florence, Unit of Oncologic Minimally Invasive Urology and Andrology, Careggi Hospital, Florence, Italy; o Department of Urology, European Institute of Oncology (IEO), Milan, Italy; p Department of Urology, La Paz University Hospital, Madrid, Spain; q Department of Urology and Oncologic Urology, Wrocław Medical University, Wroclaw, Poland; r Institute for Urology and Reproductive Health, Sechenov University, Moscow, Russia; s Karl Landsteiner Institute of Urology and Andrology, Vienna, Austria; t Department of Urology, Weill Cornell Medical College, New York, NY, USA; u Department of Urology, University of Texas Southwestern Medical Center, Dallas, TX, USA; v Department of Urology, Motol Hospital, 2nd Faculty of Medicine, Charles University, Prague, Czech Republic; w Department of Urology, CHU Bichat, Paris, France

**Keywords:** Upper tract urothelial carcinoma, UTUC, RNU, laparoscopic, radical nephroureterectomy, open

## Abstract

**Objectives:**

To compare oncological outcomes of open (ORNU) and laparoscopic radical nephroureterectomy (LRNU) after controlling for preoperative patient-derived factors.

**Patients and methods:**

We evaluated a multi-institutional collaborative database composed of 3984 patients diagnosed with upper tract urothelial carcinoma (UTUC) treated with RNU between 2006 and 2018. To adjust for potential selection bias, propensity score matching adjusted for age, gender and American society Anesthesiology (ASA) score was performed with one ORNU patient matched to one LRNU patient. Uni- and multivariable Cox regression evaluating the risk of overall recurrence, cancer-specific mortality (CSM) and overall mortality (OM) in the overall population and after propensity matching were performed.

**Results:**

In total, 3984 patients underwent RNU, of these 3227 (81%) patients were treated with ORNU and 757 (19%) patients with LRNU. Within a median follow-up of 62 months, 1276 recurrences, 844 CSMs and 1128 OMs were recorded. On multivariable analyses, the LRNU approach was associated with an increased risk of overall recurrence (hazard ratio [HR] 1.26, 95% confidence interval [CI] 1.03–1.54; *P* = 0.02), but on the other hand LRNU was associated with a protective effect on CSM (HR 0.74, 95% CI 0.56–0.98; *P* = 0.04). After propensity matching analyses adjusted for age, gender and ASA score, 757 patients treated with LRNU and 757 patients treated with ORNU were available for the analyses. On multivariable Cox regression, LRNU vs ORNU was not associated with any difference in overall recurrence (*P* = 0.08), CSM (*P* = 0.1) or OM (*P* = 0.9).

**Conclusion:**

Our present data suggest that even if the type of approach to RNU was associated with different survival outcomes considering the overall population, this difference vanished when adjusted for potential confounders in propensity matching analyses. Therefore, we found that LRNU is not inferior to the ORNU approach for the treatment of UTUC.

**Abbreviations:**

ASA: American Society of Anesthesiology; CIS: carcinoma *in situ*; CSM: cancer-specific mortality; HR: hazard ratio; IQR: interquartile range; LN: lymph node; LNI: lymph node invasion; LVI: lymphovascular invasion; OM: overall mortality; pT: pathological tumour stage; RCT: randomised controlled trial; (L)(O)RNU: (laparoscopic) (open) radical nephroureterectomy; UTUC: upper tract urothelial carcinoma

## Introduction

Upper tract urothelial carcinoma (UTUC) is an uncommon neoplasm accounting for about 5–10% of all urothelial malignancies. Radical nephroureterectomy (RNU) with bladder cuff excision, with a single postoperative dose of intravesical chemotherapy, is the standard therapy for high-risk UTUC [1–3]. Although several demographic and pathological features such as presence of lymph node (LN) metastases [4], histological variants [5,6], lymphovascular invasion (LVI) [7] or smoking status [8] have been validated in predicting survival outcomes, the safety of minimally invasive techniques remains under investigation, especially for locally advanced disease.

Laparoscopic RNU (LRNU) has been proposed in the last 10 years as an alternative technique to the open approach. However, data from a randomised controlled trial (RCT) raised the hypothesis that patients with locally advanced disease might have worse survival outcomes if treated with a laparoscopic approach compared to open RNU (ORNU) [9]. However, given the paucity of studies comparing these different approaches, data are urgently required to confirm the safety of minimally invasive techniques.

To address this unmet need, we collected complete data from a large multicentre UTUC collaboration of patients treated with RNU at academic centres to determine the impact of minimally invasive LRNU on survival outcomes compared to standard ORNU. We repeated the analyses considering only patients with locally advanced UTUC and after propensity matching to limit the impact of selection bias on survival outcomes.

## Patients and methods

The present study was approved by an Institutional Review Board for institutional data sharing from all of the participating sites of the Upper Tract Urothelial Carcinoma Collaboration [10]. Consecutive patients were treated between 2006 and 2018 with curative intent extirpative surgery in the form of RNU performed for non-metastatic UTUC. Complete clinical data were available for 3984 patients diagnosed with UTUC. The regional LNs were resected at the surgeon’s discretion. Separate analyses were performed for patients affected by pT1/2 pN0, pT3/T4 pN0, and pN+ disease.

### Variable definitions

Patient information included age at surgery, gender, American Society Anesthesiology (ASA) status, surgical technique used, pathological tumour status (pT), pathological grade, LN invasion (LNI), number of LNs removed, presence of tumour necrosis, presence of LVI, concomitant carcinoma *in situ* (CIS), and administration of adjuvant chemotherapy. LVI was considered present, when cancer cells were within an endothelium-lined space without underlying muscular walls [11].

### Primary and secondary endpoints

The primary endpoint was to compare the survival outcomes of ORNU with LRNU. The secondary endpoint was to investigate the impact of surgical technique on survival outcomes of patients with locally advanced UTUC. Overall recurrence and cancer-specific mortality (CSM) were defined as disease recurrence and death from disease, respectively.

### Statistical analyses

Descriptive statistics of categorical variables focussed on frequencies and proportions. Means, medians, and interquartile ranges (IQRs) were reported for continuously coded variables. The Mann–Whitney and chi-square tests were used to compare the statistical significance of differences in medians and proportions, respectively. Fine and Gray multivariable competing risk analyses tested the impact of surgical technique and survival outcomes. Owing to inherent differences between patients undergoing ORNU and LRNU in terms of baseline patient and disease characteristics, we used a 1:1 propensity score-matched analysis to adjust for the effects of these differences. The use of the propensity score-matching method reduces the customary bias associated with the conventional multivariable modelling approach. The variables adjusted for were age, gender, preoperative ASA score, and cT stage. Subgroup analyses were performed. Statistical significance was considered at *P* < 0.05. Statistical analyses were performed using the Statistical Package for the Social Sciences (SPSS®), version 22.0 (IBM Corp., Armonk, NY, USA) and STATA 14 (Stata Corp., College Station, TX, USA).

## Results

### Baseline characteristics

Demographics and pathological characteristics of the cohort stratified by surgical approach are shown in Table 1. Overall, 757 (19%) patients were treated with LRNU and 3227 (81%) with ORNU; 68% (*n* = 2725) of the patients were male and the median (IQR) age was 69 (61–76) years. In all, 48% of the patients (*n* = 1899) harboured pathological Stage T3–T4, 61% had high-grade disease (*n* = 2426), and 11% (*n* = 202) had LN metastases. Difference in age at surgery and gender between LRNU and ORNU patients was not statistically significant (all *P* > 0.05). Conversely, patients treated with LRNU had lower ASA scores (ASA 3–4: 27% vs 55%), lower pT3–T4 disease stage (34% vs 51%), and lower rate of high-grade disease (40% vs 66%) compared to those treated with ORNU.

**Table 1.** Baseline characteristics of patients with UTUC according to the type of surgery (ORNU vs LRNU) in the whole and matched cohorts

	Whole cohort				Matched cohort			
Variable	Overall (3984, 100%)	ORNU (3227, 81%)	LRNU (757, 19%)	*P*	Overall (1514, 100%)	ORNU (757, 50%)	LRNU (757, 50%)	*P*
**Age, years** Mean Median (IQR)	68 69 (61–76)	68 69 (61–76)	69 70 (62–77)	0.06	69 70 (62–77)	69 70 (63–77)	69 70 (62–77)	0.2
**Gender**, *n* (%) Male Female	2725 (68) 1259 (32)	2214 (89) 1013 (11)	511 (67) 246 (33)	0.6	1028 (68) 486 (32)	517 (68) 240 (32)	511 (67) 246 (33)	0.8
**ASA score**, *n* (%) 1–2 3–4	1529 (68) 721 (32)	976 (55) 517 (45)	553 (73) 204 (27)	<0.001	1102 (73) 408 (27)	549 (73) 208 (27)	553 (73) 204 (27)	0.9
**pT stage**, *n* (%) pT0–1 pT2 pT3–4	1284 (32) 801 (20) 1899 (48)	943 (29) 643 (20) 1641 (51)	241 (45) 158 (21) 258 (34)	<0.001	616 (41) 379 (25) 519 (34)	275 (36) 221 (29) 261 (35)	341 (45) 158 (21) 258 (34)	0.04
**LNI**, *n* (%)	202 (11)	162 (14)	40 (6)	<0.001	161 (11)	113 (15)	48 (6)	0.004
**Number of LNs removed** Mean Median (IQR)	3 0 (0–3)	3 0 (0–3)	2 0 (0–1)	0.001	3 0 (0–4)	4 1 (0–7)	2 0 (0–1)	<0.001
**High grade**, *n* (%)	2426 (61)	2121 (66)	305 (40)	<0.001	658 (44)	353 (47)	305 (40)	0.03
**Tumour necrosis**, *n* (%)	778 (22)	656 (22)	122 (24)	0.2	242 (21)	120 (19)	122 (24)	0.09
**LVI**, *n* (%)	931 (26)	754 (26)	177 (27)	0.3	375 (29)	198 (32)	177 (27)	0.2
**Concomitant CIS**, *n* (%)	617 (20)	534 (20)	83 (18)	0.2	191 (19)	108 (21)	83 (18)	0.3
**Adjuvant chemotherapy**, *n* (%)	475 (12)	370 (12)	105 (14)	0.1	220 (15)	115 (16)	105 (14)	0.4

### Clinicopathological characteristics (adjusted cohort)

Demographics and pathological characteristics of the cohort after propensity matching, stratified by surgical approach are shown in Table 1. Overall, 744 (50%) patients were treated with LRNU and 744 (50%) with ORNU. After propensity matching, differences between patients treated with ORNU and LRNU comprised pT stage (*P* = 0.04), LNI (*P* = 0.004), number of LNs removed (*P* < 0.001), tumour grade (*P* = 0.03), and tumour necrosis (*P* = 0.09).

### Survival analyses in the entire cohort (unadjusted cohort)

**Figure 1.**
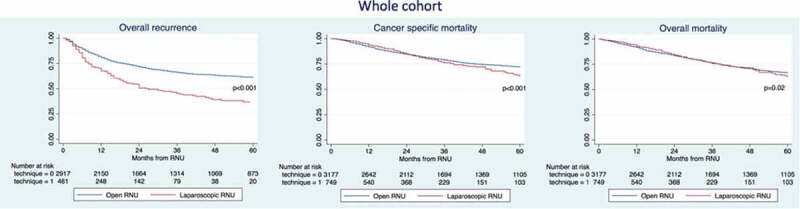
Kaplan–Meier survival analysis of overall recurrence, CSM and OM in patients treated with RNU for UTUC

The median follow-up of the entire cohort was 62 months. The 3-year recurrence rates, CSM and overall mortality (OM) were 47% vs 65%, 77% vs 81% and 75% vs 75% for LRNU vs ORNU, respectively (Figure 1). On multivariable analyses (Table 2), the LRNU approach was associated with increased risk of overall recurrence (hazard ratio [HR] 1.26, 95% CI 1.03–1.54; *P* = 0.02); but, on the other hand LRNU was associated with a protective effect on CSM (HR 0.74, 95% CI 0.56–0.98, *P* = 0.04). No differences were recorded regarding OM (*P* = 0.2).

**Table 2.** Multivariable Cox regression analyses predicting the risk of overall recurrence, CSM and OM in patients treated with RNU in the whole cohort

Variables	Overall recurrence		CSM		OM	
	HR (95% CI)	*P*	HR (95% CI)	*P*	HR (95% CI)	*P*
Age, years	1.01 (1.00–1.01)	0.001	1.02 (1.01–1.03)	<0.001	1.04 (1.03–1.04)	<0.001
Gender (male vs female)	0.97 (0.82–1.14)	0.7	0.94 (0.78–1.13)	0.5	0.88 (0.75–1.03)	0.1
LRNU vs ORNU	1.26 (1.03–1.54)	0.02	0.74 (0.56–0.98)	0.04	0.87 (0.69–1.08)	0.2
pT stage pT2 vs pT1–0 pT3–4 vs pT1–0	1.87 (1.35–2.59) 4.62 (3.47–6.16)	<0.001 <0.001	1.48 (0.94–2.34) 5.50 (3.72–8.16)	0.09 <0.001	0.78 (0.59–1.03) 1.77 (1.38–2.27)	0.08 <0.001
Presence of LNI	2.06 (1.65–2.56)	<0.001	1.53 (1.27–1.85)	<0.001	2.03 (1.62–2.53)	<0.001
Number of LNs removed	1.00 (0.99–1.01)	0.3	1.00 (0.98–1.01)	0.8	0.99 (0.98–1.00)	0.4
Presence of LVI	1.46 (1.23–1.73)	<0.001	1.53 (1.27–1.85)	<0.001	1.47 (1.25–1.73)	<0.001
Pathological grade (HG vs LG)	0.42 (0.34–0.52)	<0.001	1.01 (0.77–1.32)	0.9	1.47 (1.17–1.85)	0.001
Tumour necrosis	1.28 (1.08–1.50)	0.003	1.18 (0.99–1.43)	0.06	1.17 (1.00–1.37)	0.04
Presence of concomitant CIS	1.07 (0.90–1.26)	0.4	1.02 (0.84–1.24)	0.8	1.09 (0.93–1.28)	0.2
Presence of PSM	0.92 (0.53–1.59)	0.8	1.71 (0.94–3.13)	0.07	1.66 (0.88–3.13)	0.1
Adjuvant chemotherapy	1.63 (1.34–2.00)	<0.001	1.74 (1.40–2.16)	<0.001	1.25 (1.02–1.54)	0.03

HG, high grade; LG, low grade; PSM, positive surgical margins.

### Survival analyses after propensity matching (adjusted cohort)

**Figure 2.**
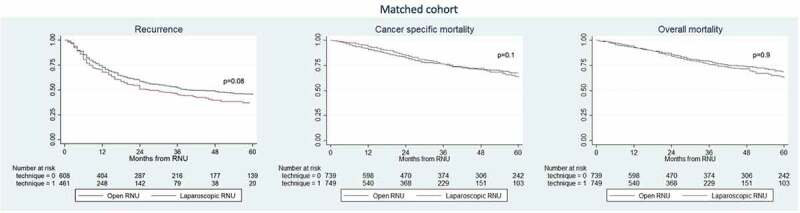
Kaplan–Meier survival analysis of overall recurrence, CSM and OM in patients treated with RNU for UTUC in the matched cohort

The 3-year recurrence rates, CSM and OM were 47% vs 42%, 75% vs 71% and 74% vs 73% for LRNU vs ORNU, respectively (Figure 1). After propensity score-matched analysis adjusted for age, gender, ASA score, cT stage, and adjuvant chemotherapy, 744 patients treated with LRNU and 744 patients treated with ORNU were available for the analyses (Figure 2). On multivariable Cox regression (Table 3), LRNU vs ORNU was not associated with any difference in overall recurrence (*P* = 0.06), CSM (*P* = 0.1) or OM (*P* = 0.9).

**Table 3.** Multivariable Cox regression analyses predicting the risk of overall recurrence, CSM and OM in patients treated with RNU after propensity matching 1:1

Variables	Overall recurrence		CSM		OM	
	HR (95% CI)	*P*	HR (95% CI)	*P*	HR (95% CI)	*P*
Age, years	1.00 (0.99–1.02)	0.5	1.03 (1.01–1.05)	0.004	1.04 (1.02–1.06)	<0.001
Gender (male vs female)	0.82 (0.60–1.12)	0.2	0.81 (0.54–1.21)	0.3	0.73 (0.51–1.05)	0.09
LRNU vs ORNU	1.33 (0.96–1.84)	0.08	0.69 (0.43–1.11)	0.1	1.01 (0.68–1.50)	0.9
pT stage pT2 vs pT1-0 pT3–4 vs pT1–0	1.75 (1.13–2.72) 3.44 (2.17–5.45)	0.01 <0.001	1.70 (0.80–3.59) 5.67 (2.74–11.75)	0.1 <0.001	0.77 (0.46–1.32) 1.66 (0.96–2.88)	0.3 0.07
Presence of LNI	2.30 (1.48–3.56)	<0.001	2.59 (1.60–4.18)	<0.001	2.49 (1.55–3.97)	<0.001
Number of LN removed	1.01 (0.99–1.03)	0.06	1.01 (0.99–1.04)	0.1	1.01 (0.99–1.03)	0.3
Presence of LVI	1.52 (1.10–2.12)	0.01	1.26 (0.81–1.96)	0.3	1.35 (0.91–2.02)	0.1
Pathologic grade (HG vs LG)	0.32 (0.23–0.45)	<0.001	0.88 (0.55–1.41)	0.6	1.53 (0.99–2.35)	0.05
Tumour necrosis	1.38 (1.01–1.92)	0.04	1.30 (0.86–1.95)	0.2	1.21 (0.83–1.76)	0.3
Presence of concomitant CIS	1.01 (0.74–1.40)	0.9	0.93 (0.61–1.42)	0.7	0.92 (0.63–1.34)	0.6
Presence of PSM	0.69 (0.35–1.36)	0.3	1.16 (0.53–2.57)	0.7	1.08 (0.43–2.70)	0.8
Adjuvant chemotherapy	0.82 (0.54–1.25)	0.4	1.27 (0.79–2.03)	0.3	0.91 (0.56–1.47)	0.7

HG, high grade; LG, low grade; PSM, positive surgical margins.

## Discussion

Although some survival differences existed considering LRNU and ORNU for the treatment of UTUC in the whole population, we found that these differences vanished after adjusting for preoperative characteristics (age, gender, ASA score, and cT stage) using a propensity score-matching analysis.

Our present results are in contrast with the only RCT designed to investigate this aspect. Simone *et al*. [9] compared 40 patients treated for UTUC with LRNU vs 40 patients treated with ORNU. They found no difference between the laparoscopic and open approach in the overall population; however, when pT3 patients only and high-grade tumour were considered, ORNU was associated with lower risk of recurrence and CSM compared to LRNU. Limitations of the study included the small sample size, the single centre experience, and the absence of concomitant lymphadenectomy. These findings were confirmed by Kim *et al*. [12] in a retrospective monocentric study, in which 271 patients were treated with ORNU and compared to 100 patients treated with LRNU. The authors found that the laparoscopic approach was associated with inferior overall survival and cancer-specific survival compared to ORNU. These results were particularly evident when considering patients with locally advanced disease. On the other hand, several reports failed to observe survival differences regarding the type of RNU. Miyazaki *et al*. [13] analysed data of 1509 patients with UTUC from 348 Japanese institutions, finding no difference regarding surgical approach in locally advanced disease. Similar results were observed by Walton *et al*. [11] in a consortium who analysed 773 patients with UTUC, observing an oncological equivalence between ORNU and LRNU. Similar findings were reported by Hanna *et al*. [14] using Nationwide Inpatient Sample database.

Several hypotheses have been proposed regarding the possible lower oncological safety of LRNU compared to ORNU. First, differences regarding the two procedures might be related to the delivery of concomitant lymphadenectomy and to the number of LNs removed [15], as LRNU seems to be associated with a lower number of LNs removed: this aspect might be important, especially when locally advanced UTUCs are considered. Second, the risk of urine spillage for UTUC might increase the risk of recurrence in laparoscopic surgery, due to pneumoperitoneum and the increasing of intra-abdominal pressure. However, these theories have never been proved, even considering patients treated with robot-assisted or laparoscopic surgery for bladder cancer that showed similar results to UTUC, especially when considering advanced disease [16]. Third, distal ureteric management might play a critical oncological role. Laparoscopic nephrectomy with open excision of the bladder cuff with distal ureter or laparoscopic excision of the bladder cuff and distal ureter might have different survival outcomes and in the only randomised trial available, all the patients were treated with laparoscopic bladder cuff [17].

The clinical implications of our present study are several-fold. Although some studies have raised doubts about the safety of oncological outcomes of LRNU compared to ORNU for patients with pT3–4 disease [9,12,17–19], in our present multicentre collaboration we found that there were no differences in survival outcomes regarding ORNU or LRNU. Our present study benefits from being a large multicentre series and propensity matching analyses minimised selection bias.

Our present study is not devoid of limitations. First and foremost, we recognise that our study is limited by its observational nature, and thus our present results should be interpreted within the limits of its retrospective design. In this regard, the decision regarding the surgical technique, the extension of lymphadenectomy or the use of perioperative chemotherapy was not standardised or randomised, but was decided upon by the treating surgeon. On the other hand, the numerosity of our present cohort and the use of propensity matching analyses reduce partially the selection bias. Second, all patients included in our present cohort underwent ORNU or LRNU at referral centres. Therefore, our present findings might not be applicable to other non-referral centres. Third, no standardised pathological review was performed, although every centre benefitted from a specialised uropathologist in evaluating the number of LNs removed, number of metastatic LNs or presence of histological variants. Fourth, no data about the type of bladder cuff excision were available in our database; therefore, no definitive conclusion can be made regarding this aspect. Fifth, not all patients with locally advanced disease were treated with adjuvant chemotherapy [20] and its delivery was based on the decision of the treating oncologist. Sixth, the learning curve is an important parameter to evaluate surgical outcomes, but unfortunately these data were not available for our present cohort.

## Conclusion

After the propensity score-matched analysis adjusted for all major confounders, no differences were found comparing ORNU and LRNU. High-quality prospective trials are warranted to support the long-term oncological safety of LRNU.
